# Robot-assisted Resection of Endobronchial Lipomatous Hamartoma with Hilar Extension

**DOI:** 10.7759/cureus.3930

**Published:** 2019-01-21

**Authors:** Jeff D Hodges, Ray Chihara, Edward Y Chan, Min Kim

**Affiliations:** 1 Surgery, Houston Methodist Hospital, Houston, USA

**Keywords:** endobronchial lipomatous hamartoma, robot assisted right lower lobe superior segmentectomy, pulmonary resection, airway reconstruction, five on a dice port placement

## Abstract

Endobronchial lipomatous hamartoma with hilar extension is a very rare benign disease of the airway. A 78-year-old male with coronary artery disease and a coronary stent presented with worsening shortness of breath. The workup included a computed tomography (CT) scan of the chest that showed a nearly obstructive lipomatous endobronchial lesion in the right bronchus intermedius with 2 cm hilar extension of the lipomatous mass. We performed a robot-assisted resection of the lipomatous mass using the “five on a dice” port placement for the Da Vinci Xi robot. In order to fully obtain the exposure necessary to resect the mass and the endobronchial lesion, we performed a right lower lobe superior segmentectomy. The margin of the parenchyma was determined using indocyanine green angiography and divided with the robot blue load stapler. The mass was carefully separated from the pulmonary artery and divided from the endobronchial portion. The frozen section showed that it was a lipomatous mass without any signs of malignancy. We then resected the endobronchial mass with robot scissors and the pathology was consistent with benign lipomatous hamartoma. Two sutures were placed as retraction sutures to pull the airway away from the pulmonary artery. We then closed the opening with absorbable sutures in an interrupted fashion. There was no air leak at the end of the case. The patient went home on postoperative day 3 without any complications and with his shortness of breath resolved. The use of the Da Vinci Xi robot allows for a successful minimally invasive method of resecting a rare endobronchial hamartoma with hilar extension.

## Introduction

Pulmonary hamartomas represent approximately 70% of all benign lung tumors. Endobronchial lesions represent a much rarer variant of the disease. Many of these endobronchial lesions are amenable to endobronchial resection [1-4], however, we present a unique case of endobronchial lipomatous hamartoma with hilar extension [5] that is not amenable for endobronchial resection. We performed a robot-assisted resection right lower lobe superior segmentectomy to provide exposure to the mass and performed resection of the endobronchial lipomatous hamartoma and hilar mass with repair of the bronchus intermedius.

## Case presentation

A 78-year-old male patient with coronary artery disease status post coronary stent placement was found to have a lung nodule on the chest radiograph at that time. The patient underwent a computed tomography (CT) scan, and bronchoscopy, and was found to have a 9 mm fatty endobronchial lesion in the bronchus intermedius above the middle lobe with 2 cm extraluminal fatty lesion into the right hilum. An endobronchial ultrasound with biopsy of the mass was performed, which showed benign bronchial epithelial cells. Since endobronchial resection of the mass would lead to a large defect in the right bronchus intermedius, the decision was made to perform robotic-assisted resection of the lesion (Video 1).

**Video 1:** Endobronchial Lipomatous Hamartoma with Hilar Extension

We used the Da Vinci Xi robot to perform resection of the endobronchial lesion and hilar mass with right lower lobe superior segmentectomy to remove the lesion. The patient had a “five on a dice” port placement for the operation [6, 7]. First, we performed the right lower lobe superior segmentectomy to obtain adequate exposure of the hilar mass. We mobilized the superior segmental branch of pulmonary artery and superior segmental branch of the right lower lobe going to the inferior pulmonary vein and divided them with the vascular robot stapler. We divided the superior segmental branch of right lower lobe bronchus with the robot blue load stapler. We used indocyanine green angiography to define the borders of the superior segment of the right lower lobe, which was divided using the robot blue load stapler. This provided access to the hilar fatty tumor, which allowed for removal of the hilar mass and subsequent resection of endobronchial lesion with scissors. The frozen section on both lesions was negative for malignancy. We confirmed complete resection with intraoperative bronchoscopy that also showed a large opening in the airway.

In order to reconstruct the airway, we placed two 3-0 vicryl stay sutures at the proximal and distal ends of the airway and placed the suture through the posterior ports to pull the airway posteriorly away from the main pulmonary artery. We closed the opening with 4-0 PDS (polydioxanone) in an interrupted fashion eight times. This provided good closure of the opening. We performed a bronchoscopy that showed no abnormalities and the air leak test demonstrated no appreciable air leaks.

The patient went home on postoperative day 3 without any complications. The final pathology report was lipomatous hamartoma.

## Discussion

Pulmonary hamartomas represent the most common benign lung tumor, however, the endobronchial variant is very rare. Many of these lesions may be resected via bronchoscopy, but the large size and hilar involvement of the tumor precludes safe removal of the tumor through an endoscopic procedure. Surgically, these tumors have been removed with thoracotomy [8, 9]. We found that using the robot allowed us to perform this complex procedure in a minimally invasive fashion.

## Conclusions

The robot allowed us to perform resection of rate endobronchial lipomatous hamartoma with hilar extension with superior right lower lobe segmentectomy and airway reconstruction in minimally invasive fashion that translated to excellent patient outcome.
